# Mediastinal Chyloma after Ascending Aortic Replacement: A Case of Delayed, Localized Chyle Leakage

**DOI:** 10.3400/avd.cr.25-00150

**Published:** 2026-05-13

**Authors:** Masato Yamakawa, Jun Yokote, Kei Kurokawa, Takaki Marutani, Hironari Shibahara, Toshikuni Yamamoto, Shinichi Ashida, Masato Mutsuga

**Affiliations:** 1 Department of Cardiovascular Surgery, Japan Community Healthcare Organization Chukyo Hospital, Nagoya, Aichi, Japan; 2 Department of Thoracic and Cardiovascular Surgery, Ogaki Municipal Hospital, Ogaki, Gifu, Japan; 3 Department of Pathology, Aichi Prefectural University School of Nursing and Health, Nagoya, Aichi, Japan; 4 Department of Cardiac Surgery, Nagoya University Graduate School of Medicine, Nagoya, Aichi, Japan

**Keywords:** chyle, aortic dissection, delayed complication

## Abstract

Graft-related complications, such as pseudoaneurysms and seromas, are rare following aortic replacement. The occurrence of localized chyle leakage is exceptionally uncommon. A 59-year-old male presented with chest pain due to an expanding 5-cm perigraft fluid collection 5 years after emergent ascending aortic replacement for acute aortic dissection. Surgical intervention was indicated as a pseudoaneurysm could not be ruled out. Intraoperatively, a chronic, localized chyle leakage (chyloma) was confirmed around the graft, showing high triglyceride and total cholesterol levels on biochemical analysis. The chyloma wall was resected and cauterized. No recurrence of perigraft fluid accumulation was observed during 5 years.

## Introduction

Ascending aortic replacement for acute aortic dissection rarely leads to graft-related complications. While perigraft seroma has been frequently reported in abdominal aortic procedures, its occurrence in the mediastinum, particularly involving chyle, is extremely rare. Various mechanisms have been postulated for seroma formation, including plasma leaching from graft porosity, inflammation, or infection.^1)^ We report a unique case of localized perigraft fluid collection consistent with chyle that developed late after ascending aortic replacement.

## Case Report

**Fig. 1:**
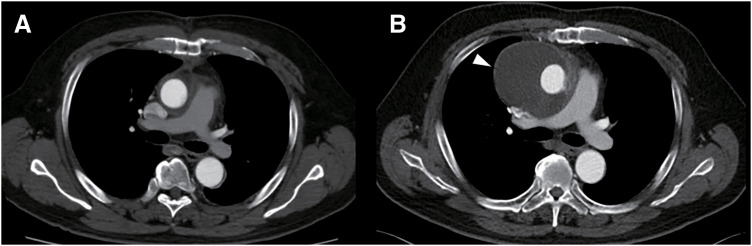
CT findings. (**A**) CT scan obtained 1 month postoperatively revealed no perigraft fluid collection. (**B**) CT scan obtained 5 years postoperatively revealed a low-density area (white arrow) suggestive of perigraft fluid collection. CT: computed tomography

A 59-year-old male with a history of hypertension and dyslipidemia presented with chest pain 5 years after undergoing emergent surgery for acute aortic dissection (DeBakey type II). At age 54, he had undergone ascending aortic replacement using a polyester knitted graft (Triplex 26 mm; Terumo, Tokyo, Japan). During the initial procedure, BioGlue (CryoLife, Kennesaw, GA, USA) was used to reinforce the proximal anastomosis. He was discharged without any postoperative complications. Two years after the surgery, routine follow-up computed tomography (CT) revealed fluid accumulation around the aortic graft, which was suspected to be a perigraft seroma. As the patient was initially asymptomatic, we opted for outpatient follow-up. Over the subsequent 3 years, the perigraft seroma gradually expanded to a diameter of 5 cm (**Fig. 1**). Contrast-enhanced CT and aortography ruled out active bleeding or blood inflow into the fluid collection area. The CT value of the low-density area of fluid was 25 Hounsfield units (HU). However, surgical treatment was planned due to the patient’s chest pain and the expanding nature of the mass.

**Fig. 2:**
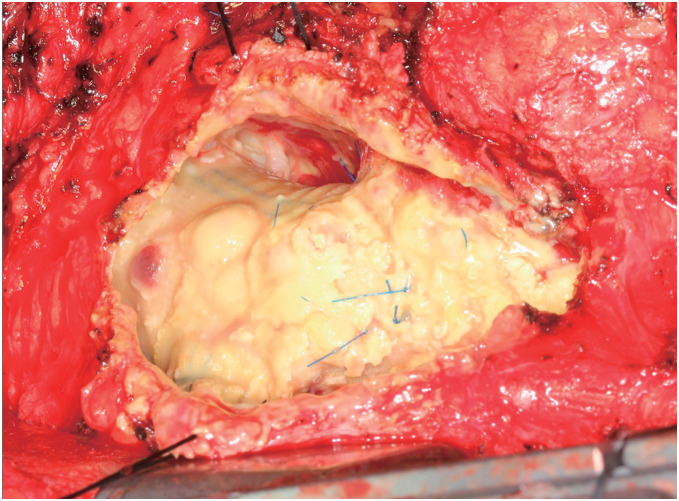
Operative findings. Intraoperative findings revealed a dissected perigraft seroma containing yellow, milky fluid that had accumulated around the aortic graft. No hematoma or bloody components were detected.

**Fig. 3:**
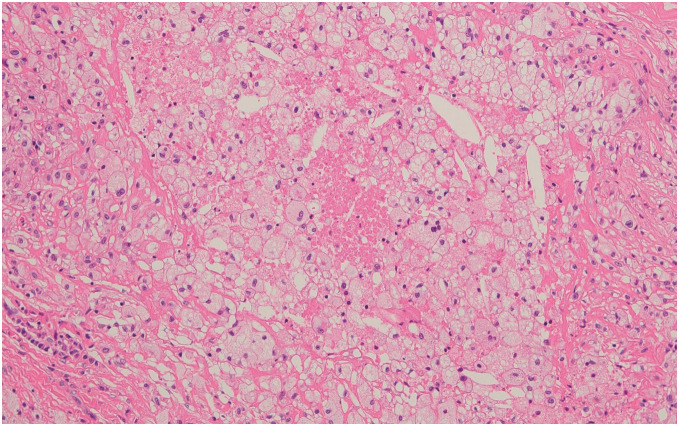
Histopathological findings. Numerous foam cells and cholesterol clefts occurred along with the infiltration of lymphocytes and plasma cells. HE stain ×100. HE: hematoxylin and eosin

Given the possibility of a pseudoaneurysm and the associated risk of rupture, we decided to use cardiopulmonary bypass (CPB). CPB was established via the right femoral and right subclavian arteries for perfusion and the right femoral vein for drainage before a median sternotomy. Intraoperative findings revealed pale-yellow, milky fluid and tissue accumulation around the aortic graft (**Fig. 2**). No macroscopic bleeding was observed from the graft or the sites of anastomosis. Intraoperative rapid tests did not confirm bacterial growth or neutrophil accumulation. We therefore diagnosed this as a localized mediastinal chyle leakage (mediastinal chyloma). Although an intraoperative fat challenge was performed, the definitive source of the chyle leakage could not be identified. All fluid was drained, and the area was irrigated. The chyloma wall was resected as much as possible, and the residual tissue was cauterized. Biochemical analysis of the fluid confirmed high lipid concentrations: triglyceride (TG): 123 mg/dL and total cholesterol (T-CHO): 790 mg/dL. Due to the high number of lipid droplets, the exact number of lymphocytes could not be determined. Intraoperative sample culture and PCR tests for acid-fast bacilli were negative. Histopathological examination revealed that the chyloma wall was composed of fibrotic connective tissue with vitrification. Inside the wall, there were numerous foam cells and cholesterol clefts with infiltration of lymphocytes and plasma cells (**Fig. 3**). No bacteria or fungi were detected by Grocott staining. These findings were consistent with inflammatory reactions to chyle. The patient’s postoperative course was stable, and no recurrence of fluid retention was observed during the 5 years of follow-up.

## Discussion

We experienced a unique case of chyle-like fluid accumulation that presented as a localized mass 5 years after ascending aortic replacement. Five possible etiologies for this condition were hypothesized.

The first is “thoracic duct injury.” While chylothorax after the ascending arch and descending aortic replacement or harvesting of the internal thoracic artery has been reported,^2,3)^ its absence in the early postoperative period makes direct traumatic injury to the main thoracic duct unlikely. A possible cause of chyle accumulation is the abnormal proliferation of lymphatic vessels.

The second is “pseudochylothorax.” Several articles report that pseudochylothorax (cholesterol pleurisy) occurs in fibrotic pleura with the accumulation of long-standing chyle-like pleural effusion. The 3 major causes of pseudochylothorax are tuberculous, traumatic, and rheumatoid etiologies. The definition of chylothorax is more than 110 mg/dL TG or 50–100 mg/dL TG with chylomicrons in the pleural effusion. On the other hand, the definition of a pseudochylothorax is more than 200 mg/dL T-CHO, and cholesterol crystals can be observed via microscopy.^4)^ In our case, the fluid data (TG: 123 mg/dL, T-CHO: 790 mg/dL) satisfied the definitions for both chylothorax and pseudochylothorax. The histological findings of foam cells and cholesterol clefts strongly support a diagnosis related to chronic inflammation induced by long-standing chyle or chyle-like fluid.

The third is “aseptic abscess/infection.” There are also some reports of aseptic abscesses caused by tuberculosis and fungi after ascending replacement.^5)^ In this case, the PCR test and culture for acid-fast bacilli were negative. Grocott staining detected no fungi, although a fungal PCR test was not performed. Aseptic abscess is incompatible with these results or with clinical and intraoperative findings.

The fourth is “BioGlue (CryoLife) reaction.” Noninfectious abscess formation related to BioGlue has been reported.^6)^ In this case, BioGlue was used in the initial surgery. However, BioGlue-related inflammatory reactions typically occur in the early postoperative phase and often present as sterile abscesses. In our case, where it took 2 years for the fluid to accumulate, this etiology was unlikely to have occurred. The late onset (5 years postoperatively), the high lipid levels in the fluid, and the characteristic pathological findings of foam cells and cholesterol clefts led us to conclude that the mass was a chyloma rather than simple BioGlue reactions.

The fifth is “perigraft seroma.” Perigraft seroma after ascending or ascending arch replacement for acute aortic dissection is often reported,^7,8)^ and the specific histological findings of foam cells and cholesterol clefts in our case distinguished it from a typical inflammatory seroma. Although the Triplex graft is a zero-porosity sealed graft designed to minimize plasma leakage and is generally considered resistant to seroma formation,^9)^ the fluid in this case was confirmed to be chyle, not serum.

Based on the above findings, we conclude that the localized fluid collection represented an encapsulated chyloma (mediastinal chyloma) originating from chronic, low-volume lymphatic leakage. In ascending aortic procedures, injury to the main thoracic duct is uncommon. However, disruption of the right lymphatic duct or minor lymphatic vessels near the thymus and innominate vein can occur during surgery. While specific patient-related factors contributing to this complication remain unclear after thorough review of the clinical data, we hypothesize that a slow, low-volume leak from these minor vessels was contained by postoperative adhesions, gradually forming an encapsulated chyloma over 5 years. The CT value of the fluid was 25 HU. While typical chyle shows lower attenuation due to its fat content, this higher value likely reflects the chronic concentration of chylous components, persistent fibrin exudation, and the accumulation of inflammatory debris. If the lesion had consisted solely of foam cells like xanthoma, it might have appeared as lower density on CT. Although the CT value was atypical, we could not rule out the possibility of a pseudoaneurysm due to the patient's chest pain and expanding fluid retention. Consequently, chyle leakage was not included in the differential diagnosis, and surgical intervention was performed without preceding lymphangiography. In this case, the patient’s chest pain and the progressive expansion of the mass led us to prioritize surgical intervention. If the patient had been asymptomatic and chyloma had been suspected preoperatively, conservative management, such as dietary fat restriction or octreotide, might have been considered. To prevent such rare complications, meticulous cauterization or ligation of all fatty tissues and potential lymphatic vessels near the innominate vein and thymus is essential, even during emergent procedures.

Surgical exploration allowed us to rule out a pseudoaneurysm and provide definitive treatment by resecting the fibrous wall and cauterizing the residual tissue. For chyle leakage of an unknown source, indocyanine green staining of the lymphatic vessels and cauterization of the leakage site have been reported.^10)^ Our procedure was effective, although the precise source of chyle accumulation remains undetermined.

## Conclusion

We report a rare case of an expanding mediastinal chyloma occurring 5 years after ascending aortic replacement. This delayed, localized chronic chyle accumulation, mimicking an aortic mass, was effectively treated by surgical resection and cauterization of the chyloma wall. No recurrence was observed on the 5-year postoperative CT scan. Further case collection is necessary to fully elucidate the pathophysiology and guide the appropriate diagnosis and treatment of this specific, delayed complication.
